# Management and Outcomes of Congenital Atrioventricular Block in Neonates: A 6-Year Experience at a Tunisian Tertiary Center

**DOI:** 10.1055/a-2616-5273

**Published:** 2025-06-09

**Authors:** Rim B. Aziza, Sameh Hajji, Khaoula Samaali, Hajer Chourou, Wafa B. Ammar, Neila B. Aba, Khaled Neji, Samia Kacem

**Affiliations:** 1 Neonatal Intensive Care Unit, Maternity and Neonatology Center of Tunis, University of Tunis El Manar Faculty of Medicine of Tunis, Tunisia; 2 University of Tunis El Manar Faculty of Medicine of Tunis, Tunisia; 3 Department of Obstetrics and Gynecology B, Maternity and Neonatology Center of Tunis, University of Tunis El Manar Faculty of Medicine of Tunis, Tunisia

**Keywords:** prenatal diagnosis, congenital atrioventricular block, echocardiography, pacemaker

## Abstract

**Background:**

Congenital atrioventricular block (CAVB) is a rare and serious condition often associated with maternal autoimmune diseases or congenital heart defects. This study aims to evaluate the clinical presentation, management, and outcomes of neonates diagnosed with CAVB.

**Methods:**

We conducted a retrospective study from January 2018 to December 2024, including eight neonates diagnosed with CAVB. Data on demographics, clinical features, treatment, and follow-up were analyzed.

**Results:**

All cases were prenatally diagnosed between 20 and 25 weeks of gestation, with positive anti-SSA/SSB antibodies in five cases. Management included cesarean delivery, Holter ECG monitoring, and pacemaker implantation for four patients. One case resulted in intrauterine fetal death, and two patients died in the neonatal period. Survivors had successful pacemaker implantation with an average follow-up of 18 months.

**Conclusion:**

Early prenatal detection and timely management of CAVB are crucial. Pacemaker implantation significantly improves survival, though challenges such as resource limitations and the lack of long-term follow-up data remain. Future studies should address these gaps to optimize care for CAVB patients.

Congenital atrioventricular block (CAVB), identified when diagnosed during fetal life, at birth, or within the first month postpartum, is a rare condition with an estimated incidence of 1 in 15,000 to 1 in 22,000 live births. 1 It is a conduction disorder characterized by a delay and/or interruption of the electrical impulse between the atria and the ventricles due to a structural or functional abnormality. 1 2 It is a rare but serious condition due to its potentially life-threatening nature during the antenatal or neonatal period. CAVB can present either as an isolated atrioventricular (AV) block in a structurally normal heart or in association with congenital heart defects, the latter carrying a worse prognosis. 3 Based on its incidence and the current understanding of its pathophysiology, CAVB can be categorized as immune-mediated or autoimmune, associated with congenital heart defects, or idiopathic. 3 4 5 The most common form of CAVB is autoimmune, typically diagnosed in children born to mothers who have, in most cases, systemic lupus erythematosus (SLE) or Sjögren's syndrome (SS) where the transplacental passage of maternal antibodies induces fetal “myocarditis,” leading to fibrosis of the conduction tissue. 6 7 Echocardiography remains the gold standard for diagnosis of CAVB, with a diagnostic accuracy of 90%. 1 The severity of CAVB depends on the block type, its onset speed, and the resulting escape rhythm. Cardiac pacing is recommended for symptomatic patients and has several prophylactic indications for asymptomatic patients to prevent sudden death. 3

Considering the rarity of this congenital condition and the conflicting data in the literature regarding its treatment and prognosis, we believe it is essential to share our findings. This article presents cases from our clinical experience, highlighting their immediate and mid-term outcomes.

## Methods

We conducted a monocentric observational retrospective study over 6 years (January 1, 2018–December 31, 2024) in the Neonatology department of The Maternity and Neonatology Center of Tunis. We collected all cases diagnosed with CAVB. Demographic, clinical, and echocardiographic data at diagnosis, as well as follow-up data and therapeutic approaches, were recorded retrospectively.

Medical investigations were performed in line with the principles of the Declaration of Helsinki. Approval was obtained from the Ethical Committee of Maternity and Neonatology Center of Tunis (November 2024). Informed consent was obtained from all the participants' parents included in the study.

## Results

We collected eight cases of CAVB with a gender ratio of 1, consisting of four boys and four girls. Consanguinity was noted in two patients. A family history of autoimmune disease was reported in two cases: a case of maternal SLE and one case of paternal autoimmune thrombocytopenia and maternal type 1 diabetes. For the other five cases, there were no maternal infections, diabetes, or alcohol use during pregnancy, and no clinical signs of maternal connective tissue disease. Additionally, there was no family history of congenital heart disease or recurrent pregnancy loss. CAVBs were antenatally detected between 20 and 25 weeks of gestation. In all cases, obstetrical ultrasound during pregnancy detected an abnormally low heart rate, prompting referrals for fetal echocardiography for further evaluation. Two-dimensional echocardiography identified situs inversus and aortic stenosis in one case. Hydrops fetalis was observed in one case, leading to intrauterine fetal death (IUFD) as a result of poorly tolerated CAVB. Fetal echocardiographic monitoring was conducted monthly until birth for the other seven cases.

During the antenatal period, all women underwent testing for anti-SSA and anti-SSB antibodies, which returned positive in five cases.

Following a multidisciplinary consultation among obstetricians, neonatologists, and pediatric cardiologists, all deliveries were scheduled at a level 3 maternity unit. Only one patient was born prematurely, while the others were carried to term. All deliveries were performed by the cesarean section (CS) due to signs of fetal distress. After birth, an infant presented with short stature, short ribs, and polydactyly, raising suspicion of Ellis–Van Creveld syndrome. Among the five patients born to mothers positive for anti-SSA and anti-SSB antibodies, there were no cutaneous manifestations of neonatal lupus syndrome (NLS), hepatosplenomegaly, or abnormal liver function tests.

The clinical characteristics of the cases are presented in Table 1 .

**Table 1:** Clinical characteristics of the cases

Case number	Sex	Gestational age at diagnosis (wk)	History of autoimmune disease	anti-SSA and anti-SSB antibodies	Hydrops	Heart rate at birth (bpm)	Postnatal echocardiography and electrical data	Clinical signs
**1**	F	20	No	Negative	No	50	Complete CAVB; dilated left ventricle	PAH
**2**	M	25	No	Negative	No	52	CAVB mobitz type II; situs inversus, a large ASD, AS, TAPVR	Heart failure; Ellis–Van Creveld syndrome (dwarfism, short ribs, polydactyly)
**3**	M	24	No	Positive	No	60	Pericardial effusion	Asymptomatic
**4**	F	24	SLE	Positive	No	50	Complete CABV; PAH	Heart failure; PAH
**5**	M	20	No	Positive	No	60	Complete CAVB; dilated left ventricle	Heart failure
**6**	F	20	No	Positive	No	63	Complete CAVB; PAH	PAH
**7**	F	20	Autoimmune thrombocytopenia; hypothyroidism; type 1 diabetes	Positive	No	65	Complete CAVB; pericardial effusion	Asymptomatic
**8**	M	20	non	Negative	Yes; intrauterine fetal death	–	–	–

Abbreviations: ASD, atrial septal defect; AS, aortic stenosis; bpm, beats per minute; CAVB, congenital atrioventricular block; PAH, pulmonary arterial hypertension; TAPVR, total anomalous pulmonary venous return.

All newborns underwent 24-hour Holter ECG monitoring within the first 48 hours of life using the GE SEER 1000. This confirmed the prenatal diagnosis of CAVB in all cases. The recordings assessed baseline ventricular rate, rhythm variability, and potential arrhythmic events.

Among the eight cases, ventricular escape rates ranged from 50 to 65 bpm, with no evidence of spontaneous rhythm improvement. Three newborns exhibited intermittent junctional rhythms, while two had occasional atrial ectopic beats without conduction. No episodes of sustained ventricular tachycardia or pauses exceeding 3 seconds were detected.

An echocardiogram was conducted for all patients, revealing pericardial effusion in one case, pulmonary hypertension (PAH) in three cases, mildly dilated left ventricle in two cases, persistent ductus arteriosus in two cases, and a complex cardiac anomaly in one patient, characterized by situs inversus, a large patent atrial septal defect, aortic stenosis and a total anomalous pulmonary venous return (TAPVR).

Therapeutically, three newborns required treatment with furosemide and an angiotensin-converting enzyme inhibitor, while another patient was administered nitric oxide ( Table 2 ). Additionally, three patients were intubated and ventilated using Invasive Assisted Controlled Ventilation due to heart failure. Four patients were urgently transferred to the pediatric cardiology department for pacemaker placement. One patient was discharged with plans for follow-up. Two patients, one girl and one boy, did not survive. The girl passed away on her third day of life, and the boy, suspected to have Ellis–Van Creveld syndrome, died at 2 months of age.

**Table 2:** Therapy and outcome of the cases

Case number	Intrauterine treatment	Postnatal treatment	Outcome
**1**	None	Nitric oxide	Monitoring and pacemaker implant at 15 mo of age
**2**	None	Invasive assisted controlled ventilation; dobutamine; furosemide	Deceased at the age of 2 mo due to heart failure
**3**	None	None	Monitoring and pacemaker implant at 13 mo of age
**4**	None	Invasive assisted controlled ventilation; dobutamine; ACE inhibitor; furosemide	Deceased at the age of 3 d due to heart failure and refractory PAH
**5**	None	Invasive assisted controlled ventilation; dobutamine; furosemide; ACE inhibitor	Pacemaker at 20 d of age
**6**	None	None	Monitoring and pacemaker implant at 5 mo of age
**7**	None	None	Monitoring and pacemaker implant at 3 mo of age
**8**	None	–	Intrauterine fetal death

Abbreviation: ACE, angiotensin-converting enzyme.

## Discussion

CAVB has a prevalence of 1 in 15,000 to 20,000 live births. 1 3 It is considered congenital if diagnosed in utero, at birth, or within the first month of life, while childhood AV block is diagnosed between the first month and 18 years of age. Both congenital and childhood AV block can result from various etiologies and may occur in a structurally normal heart or in association with congenital heart disease. 3 7

Based on its incidence and current understanding of its pathophysiology, CAVB can be classified as immune-modulated or autoimmune, CAVB, diagnosed before birth or very early on, is considered a model of passively acquired autoimmunity. It is strongly associated with the presence of anti-SSA/Ro and/or anti-SSB/La antibodies in the mother's serum, regardless of whether she has SLE, SS, or is completely asymptomatic. 6 8 In our study, anti-SSA and anti-SSB antibodies in the mother's serums were positive in five cases, with only one patient having a prior diagnosis of lupus. In fact, in most cases, AV block occurs in fetuses of healthy mothers who are silent carriers of anti-Ro/SSA antibodies. 3 As a result, the diagnosis of maternal seropositivity is often established only after congenital AV block has been detected in the infant. This suggests that the presence of anti-Ro/SSA antibodies alone may not necessarily indicate underlying maternal pathology, highlighting the need for careful monitoring and evaluation in pregnancies where these antibodies are identified. 9

Congenital heart malformations account for 14 to 42% of CAVB cases, with L-transposition of the great arteries being the most frequently associated defect. 1 3 10 In our series, echocardiography revealed a complex heart defect, including situs inversus and a large septal defect, in one patient. Notably, heart block occurs in one-third of fetuses with heterotaxy syndrome and left atrial isomerism, significantly contributing to perinatal mortality. 3

In our series, all eight cases of CAVB were diagnosed prenatally between 20 and 25 weeks, aligning with the timeline reported in the literature. 1 3 6 11 A comprehensive diagnostic protocol combining M-mode and pulsed Doppler examinations is essential to distinguish CAVB from other arrhythmias, such as blocked atrial extrasystoles. 3 6 Echocardiography also plays a crucial role in detecting anatomical abnormalities, and signs of fetal heart failure or hydrops fetalis. 4 9 In our series, one case of hydrops fetalis resulted in IUFD due to severe CAVB at an early stage of pregnancy.

Weekly fetal echocardiographic monitoring is advised until the 24th week, transitioning to biweekly monitoring until delivery. 1 In our context, prenatal echocardiographic monitoring was conducted monthly, and managed by the pediatric cardiology unit in Tunis, the capital's sole specialized center. Despite resource limitations, this monthly follow-up allowed for the timely detection of structural and rhythm-related complications, ensuring appropriate prenatal care.

In our series, none of the patients received prenatal treatment, a decision made in consultation with pediatric cardiologists. Congenital AV block without treatment is associated with fetal and neonatal mortality rates ranging from 14 to 34%, with key risk factors including fetal hydrops and ventricular escape rates below 55 bpm. 3 12 In our cohort, case eight resulted in IUFD. This raises the question of whether prenatal intervention could have improved the prognosis.

Prenatal treatment for CAVB remains a topic of debate, with no clear consensus on the optimal approach. Suggested therapies include corticosteroids, β-adrenergic receptor agonists, hydroxychloroquine, plasmapheresis, and intravenous immunoglobulin. 13 14 Concerns about maternal and fetal side effects of antenatal corticosteroids further complicate their use. Advanced techniques like in utero percutaneous pacing remain experimental and carry significant procedural risks, including fetal deaths occurring shortly after the intervention in a notable proportion of cases. 1 15 Consequently, in most cases, pregnancies with CAVB are managed expectantly, with treatment deferred until after birth (I/a). 1 3 15

In our cohort, all deliveries were performed via CS due to signs of fetal distress, primarily bradycardia. While vaginal delivery remains the preferred mode of delivery in most cases, CS is frequently performed in high-risk pregnancies, particularly those complicated by maternal autoimmune disease. Postnatally, electrocardiography (ECG) serves as the primary diagnostic tool for identifying and characterizing heart blocks, providing crucial information about their level and nature. In our study, all newborns underwent Holter ECG monitoring within the first 48 hours of life. 16 17 18 PAH was detected in three cases, consistent with previous findings that link PAH to increased morbidity. 17 Additionally, the persistent ductus arteriosus observed in two neonates underscores the importance of close monitoring to prevent potential complications. 1 3

After birth, a heart rate <70 bpm can be managed initially with medications such as isoprenaline, atropine, and epinephrine, either alone or in combination with transcutaneous pacing and/or temporary cardiac pacing, to prevent sudden death. 1 However, despite all patients in our study presenting with low heart rates at birth, including three cases of heart failure, isoprenaline was not administered, as this medication has not been available in Tunisia for several years.

Regarding the specific management of this pathology, permanent pacemaker implantation is necessary for all cases of symptomatic, irreversible AV node disease. 3 Pacing should also be considered for asymptomatic patients with high-degree AV block who present with specific risk factors.

This intervention significantly enhances long-term survival and alleviates symptoms such as presyncope and syncope, even in asymptomatic patients. However, this procedure presents significant technical challenges, with complications arising from factors such as the patients' small size, rapid growth, and the presence of underlying cardiac malformations. 6 19 In the Tunisian context, unfortunately, the required equipment is not always available and must be imported with approval from the national social security funds, explaining the delays in implantation for our cases, even for the most urgent ones.

As highlighted in the methodology section, the small sample size restricts the ability to draw statistically significant conclusions and limits the generalizability of findings regarding the effectiveness of treatments and patient outcomes. However, given the rarity of CAVB, our study provides valuable insights into its management in a resource-limited setting.

The study's retrospective design introduces potential biases, particularly selection bias. Cases included in the study were identified from a single tertiary center, which may not fully represent the broader population of neonates affected by CAVB in Tunisia. Additionally, missing or incomplete data in patient records may have affected the accuracy of some variables. While we documented short-term outcomes, including survival after pacemaker implantation, the long-term impact of CAVB on neurodevelopmental and cardiac function remains unknown. Future prospective studies with extended follow-up are necessary to assess growth, quality of life, and late cardiac complications in affected patients.

## Conclusion

This study underscores the importance of early prenatal detection of CAVB, particularly in pregnancies complicated by maternal autoimmune diseases. Timely management, including cesarean delivery for fetal distress and postnatal pacemaker implantation, has improved short-term survival in this cohort. Despite these interventions, challenges remain in terms of resource limitations, such as the delayed availability of pacemakers in Tunisia, and the lack of long-term follow-up data to evaluate sustained outcomes. Future studies should aim to address these gaps, optimize treatment strategies, and further explore the role of prenatal interventions to improve long-term patient outcomes. The findings also emphasize the need for a comprehensive, multidisciplinary approach to managing high-risk pregnancies complicated by CAVB.
